# Feasibility and safety of laparoscopic radical cystectomy for male octogenarians with muscle-invasive bladder cancer

**DOI:** 10.1186/s12885-024-11816-7

**Published:** 2024-01-31

**Authors:** Dong-liang Pan, Lu-fang Zhang, Xiao-jian Li, Ke-ping Zhang, Peng-fei Gao, Bing Yang, Ning-chen Li

**Affiliations:** 1 Department of Urology, Peking University Shougang Hospital, Beijing, 100144 China; 2 Department of Urology, Weifang People’s Hospital, Weifang, Shandong 261041 China

**Keywords:** Bladder carcinoma, Laparoscopic radical cystectomy, Cutaneous ureterostomy, Octogenarian

## Abstract

This study was designed to evaluate the safety and feasibility of laparoscopic radical cystectomy (LRC) for male octogenarian patients with muscle-invasive bladder cancer (MIBC). Briefly, a total of 57 male octogenarian patients (A group) with bladder carcinoma were enrolled and underwent LRC and intracorporeal pelvic lymph node dissection with bilateral cutaneous ureterostomy from May 2016 to December 2022. Besides, 63 male patients (age < 80 years old) with bladder carcinoma undergoing LRC and 17 octogenarian male patients with bladder carcinoma undergoing open radical cystectomy (ORC) were enrolled in B and C groups as control. All perioperative clinical materials and outcomes of long-term follow-up, and complication were collected. The specific results were shown as follows. Compared with C group, the operation time and resected lymph node in A group was increased, and the estimated blood loss, the number of transfusion needed, duration of pelvic drainage and hospital stay after surgery was decreased. The death rate and ileus complication rate were higher in A group (12 cases) than in C group (15 cases). The cases of ureteral stricture in A group (13 cases) was decreased compared with that in C group. Overall, LRC and bilateral cutaneous ureterostomy are safe, feasible and better choices for the treatment of male octogenarian patients with MIBC. The octogenarian receiving cutaneous ureterostomy heals slowly and exists certain incomplete intestinal obstruction after surgery.

## Introduction

Radical cystectomy (RC) is the standard treatment for localized muscle-invasive bladder cancer (MIBC) [1, 2]. In the past, RC was advised for the treatment of patients who had MIBC M_0_, N_0_-N_x_, T_2_-T_4a_ [2]. Other indications included Bacille Calmette-Guérin vaccine (BCG)-refractory, BCG-unresponsive and BCG-relapsing T1G3 tumors, high risk and recurrent non-muscle-invasive tumors, as well as extensive papillary disease that is uncontrollable with transurethral resection of bladder tumor (TURB) and intravesical therapy alone. Salvage cystectomy is recommended for treating patients who do not respond to conservative therapy, suffer a recurrence following bladder-sparing treatment, and have non-urothelial carcinoma (UC). Also, MIBC patients have received RC after receiving neoadjuvant chemotherapy [3].

Although the operating time of laparoscopic RC (LRC) is longer than that of open RC (ORC), it is characterized with advantages such as less blood loss and analgesic use, fewer blood transfusions and overall complications, and a shorter hospital stay [4]. Despite better oncological outcomes shown in this study, the overall survival (OS), cancer-specific survival (CSS) and recurrence-free survival (RFS) rates following LRC appear comparable to those reported in ORC series [5]. Another study also reported that the 90-d Clavien-graded complication rates among LRC, ORC and robot-assisted RC (RARC) were not significantly different [6]. In a previous study, a small single-centre randomized controlled trial (RCT) was performed to compare laparoscopic (*n* = 19), robotic (*n* = 20), open (*n* = 20) cystectomy [7]. ORC (70%) showed a much higher 30-day complication rate than LRC (26%). As for the 90-day Clavien complication rate, it was not significantly different among the three groups [7].

Urinary diversion and RC are the two steps of the whole operation in RC. The literature consistently mentions RC complications but disregards the fact that most of them are diversion related [8]. Along with the preference and social support of patients, essential factors including cognitive function, pulmonary and cardiac function, and comorbidity need to be taken into account. Before surgery, the risk of post-operative complications can be evaluated by the American Society of Anesthesiologists (ASA) score. Major complications [9, 10], especially those associated with the urinary diversion types [11], are more likely to occur when ASA scores equal to or exceed 3 points in the BC setting. In the event that reconstructive surgery puts the patient at an unacceptably high risk (as judged by comorbidities and age), both an ileal conduit and an orthotopic neobladder should be taken into consideration. Neobladder reconstruction is often not suitable for patients over 80 years of age. The precise age for a severe contraindication is unknown, though. As for male octogenarian patients with MIBC, the safety and feasibility of LRC and urinary diversion still need to be further evaluated. In this study, male octogenarian patients with MIBC were treated with LRC combined with bilateral cutaneous ureterostomy, and the safety and efficacy of LRC were further analyzed.

## Materials and methods

### Patients

The patients diagnosed with MIBC were included in the retrospective study. A total of 57 patients who received LRC and intracorporeal pelvic lymph node dissection combined with bilateral cutaneous ureterostomy were assigned to A group. The patients in A group needed to meet the following requirements simultaneously: (1) male; (2) age ≥ 80 years old; (3) muscular invasive bladder UC; (4) LRC; (5) urinary diversion method: ureterostomy. The exclusion criteria were listed as follows: (1) female; (2) age < 80 years old; (3) non-muscle invasive or non-urothelial bladder cancer or sarcoma; (4) any bladder cancer operation method other than LRC; (5) urinary diversion method except ureterostomy.

In addition, B group and C group were established as control groups. The inclusion criteria were displayed as follow: (1) male; (2) age < 80 years old (B group); age ≥ 80 years old (C group); (3) muscular invasive bladder UC; (4) bladder cancer operation method: LRC (B group); ORC (C group); (5) urinary diversion method: ureterostomy. The patients were excluded if they met any of the following conditions: (1) female; (2) non-muscle invasive or non-urothelial bladder cancer or sarcoma; (3) any urinary diversion method except ureterostomy. According to the above criteria, 63 patients were enrolled in B group and 17 patients in C group.

All patients underwent operations at Peking University Shougang Hospital and Weifang People’s Hospital from May, 2016 to December, 2022. The operations obtained the informed consent of patients and the ethical approval of Peking University Shougang Hospital (IRB-SOP-21-00). A database was prospectively populated with the data of all patients (Table 1).

**Table 1:** Comparison of characteristics and outcomes of patients in three groups

Parameter	A group (age ≥ 80, LRC)	B group (age < 80, LRC)	C group (age ≥ 80, ORC)	H/χ^2^	* P*
Total number of patients	57	63	17		
Age	84 (82–85)	66 (62–71)^*^	82 (81–82)	105.300	< 0.001
BMI (kg/m^2^)	23 (21–25)	25 (23–25)^*^	23 (20–24)	13.368	0.001
Previous abdominal operations	7.0% (4/57)	9.5% (6/63)	11.8% (2/17)	0.451	0.798
Hb (g/L)	110 (103–117)	112 (106–121)	107 (102–110)	8.548	0.014
Comorbid conditions					
Hypertension	71.9% (41/57)	60.3% (38/63)	88.2% (15/17)	5.344	0.069
Coronary heart disease	57.9% (33/57)	49.2% (31/63)	52.9% (9/17)	0.908	0.635
COPD	7.0% (4/57)	7.9% (5/63)	64.7% (11/17)^*^	39.106	< 0.001
Diabetes	19.3% (11/57)	25.4% (16/63)	35.3% (6/17)	1.942	0.379
ASA score				4.803	0.091
2	59.6% (34/57)	52.4% (33/63)	29.4% (5/17)		
3	40.4% (23/57)	47.6% (30/63)	70.6% (12/17)		
Clinical stage				22.476	< 0.001
T2NxM0	75.4% (43/57)	74.6% (47/63)	17.6% (3/17)^*^		
T3NxM0	24.6% (14/57)	25.4% (16/63)	82.4% (14/17)^*^		
TURBT pathology				0.317	0.853
G2	22.8% (13/57)	23.8% (15/63)	29.4% (5/17)		
G3	77.2% (44/57)	76.2% (48/63)	70.6% (12/17)		
Operation time (min)	210 (195–215)	210 (200–220)	110 (100–120)^*^	45.594	< 0.001
Estimated blood loss (ml)	210 (195–230)	210 (210–230)	220 (210–600)^*^	7.370	0.025
Transfusion needed (n)	0	0	29.4% (5/17)^*^	18.937	< 0.001
Resected lymph node (n)	23 (21–26)	23 (20–26)	13 (12–15)^*^	43.669	< 0.001
Cystectomy pathology				25.988	< 0.001
G3 pT2aN0	24.6% (14/57)	19.0% (12/63)	0^*^		
G3 pT2bN0	57.9% (33/57)	60.3% (38/63)	17.6% (3/17)^*^		
G3 pT3aN0	17.5% (10/57)	20.6% (13/63)	82.4% (14/17)^*^		
Duration of pelvic drainage (day)	12 (10–14)	5 (5–5)^*^	16 (15–18)^*^	115.106	< 0.001
Hospital stay after surgery (day)	8 (8–9)	7 (7–7)^*^	14 (14–16)^*^	112.453	< 0.001
Adjuvant chemothera	0	15.9% (10/63)^*^	0	11.982	0.001
Follow-up duration (month)	64 (54–73)	66 (56–73)	45 (35–51)^*^	24.402	< 0.001
BC relapse	0	3.2% (2/63)	47.1% (8/17)^*^	27.778	< 0.001
Death					
BC	0	3.2% (2/63)	41.2% (7/17)^*^	23.320	< 0.001
Other	5.2% (3/57)	3.2% (2/63)	29.4% (5/17)^*^	10.860	0.003

*Abbreviation*: *LRC* Laproscopic radical cystectomy, *ORC* Open radical cystectomy, *Hb* Hemoglobin, *BMI* Body Mass Index, *COPD* Chronic obstructive pulmonary diseases, *ASA* American Society of Anesthesiologists, *TURBT* Transurethral bladder tumor resection, *BC* Bladder cancer

*P < 0.05 vs. A group

A post-transurethral resection of bladder was performed on each patient. A staging computed tomography (CT) scan proven and tumour pathologically proven organ confined cT_2 − 3_N_x_M_0_ high-grade bladder UC. TNM classification of the Union for International Cancer Control was used to classify the cancer stages [12]. Neither chemotherapy nor radiotherapy had ever been administered to any of the patients. The ASA physical status classification system was applied to evaluate the physical status of patients. The preoperative examinations they experienced included lung function test, chest radiography, echocardiography, routine laboratory test, CT, or magnetic resonance imaging of urinary system. Besides, the common comorbidities consisted of chronic obstructive pulmonary disease (COPD), hypertension, coronary artery disease, and diabetes mellitus.

### Surgical procedures

Prior to surgery, all patients began to take semi-liquid diet for 48 h, switched to liquid diet for 24 h, and were given oral sodium phosphates solution (Dilute 90 mL sodium phosphate solution to 1500 mL, and take orally in two doses). Patients with long-term constipation were given glycerol enema (110 mL) by the anus to assist defecation. All patients were prohibited from diet for 12 h before surgery. A gastric tube was inserted before entering the operating room. During the induction of anesthesia, a broad-spectrum systemic antibiotic was administered intravenously to each patient.

**Fig. 1:**
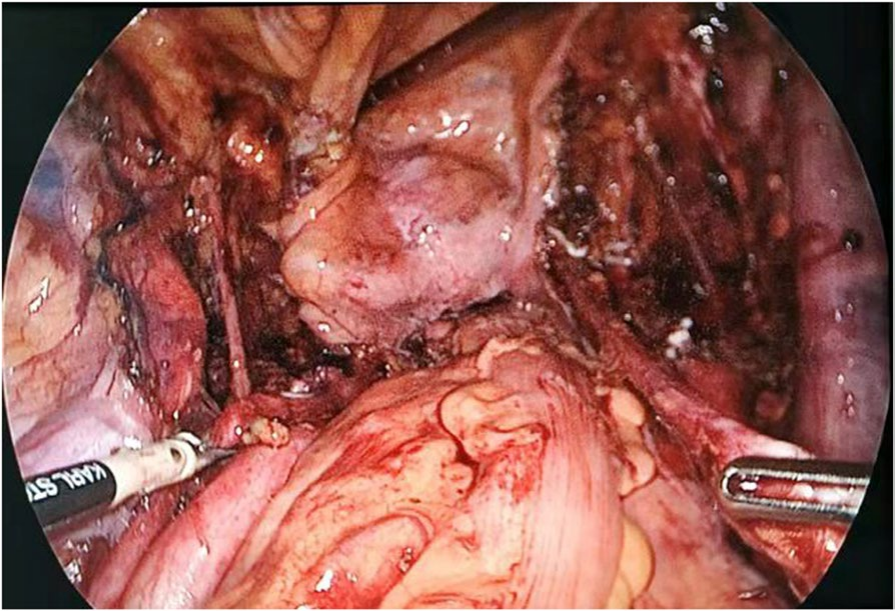
After pelvic lymphadenectomy

**Fig. 2:**
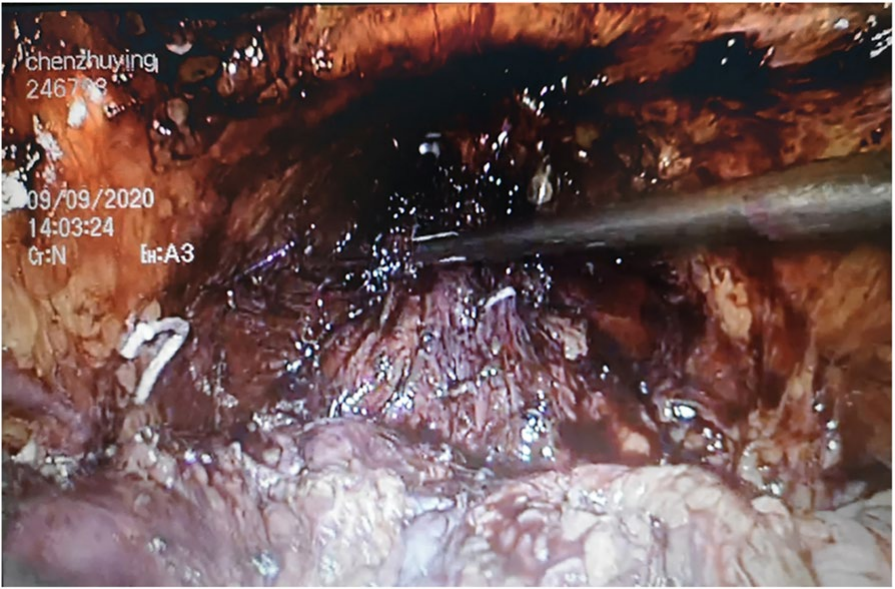
After laparoscopic radical cystectomy

The basic procedures of LRC were executed based on Campbell-Walsh Urology [13]. LRC was performed using a five-port fan-shaped trans-peritoneal approach with two 5-mm and three 10-mm trocars. The camera port was put right below the umbilicus. The formation of the pneumoperitoneum was followed by the endoscopic control of the remaining four ports. Additionally, bipolar scissors, the harmonic scalpel, and metal and plastic clips were used to dissect the tissue and ligate the vessels. The anatomical landmarks in the pelvis, like vas deferens, spermatic cord, obliterated urachus, and obliterated umbilical arteries, were located by visualizing the pelvis and loosening adhesions of the sigmoid colon during surgery. First of all, resacral, common iliac, internal, external, obturator, para-caval and para-aortic lymph nodes were all excised for pathological analysis (Fig. 1). After that, we began dissecting the seminal vesicles and posterior surface of the prostate. Then, the Retzius space, the pelvic fascia and the prostate apex and urethra were dissected in sequence. After laparoscopic cystectomy (Fig. 2), bilateral cutaneous ureterostomy was conducted. All LRC surgeries were performed by the same surgeon.

**Fig. 3:**
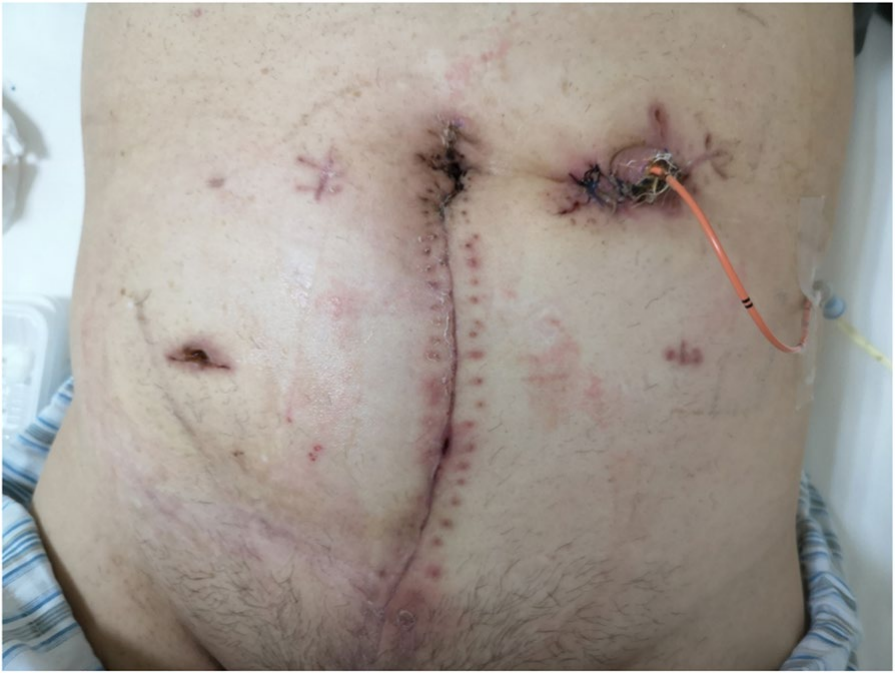
After open radical cystectomy

The steps of ORC procedure were shown as follows. Firstly, the lower abdomen underwent an incision. Then, the abdominal cavity was opened and exposed. The posterior peritoneum was cut open at the right and left iliac vessels and extended towards the pelvic floor. The left and right ureters were severed close to the bladder after being freed to the outer wall of the bladder. The proximal ureter was inserted a stent for urine drainage. Upon complete resection, lymph nodes at the obturator site, internal iliac artery, external iliac artery and vein, and the left and right common iliac artery were cleaned and removed. The anterior wall of the bladder was separated from the pubic prostate ligament, the posterior side from the denonvillier fascia to the tip of the prostate, and the lateral side of the bladder from the external iliac artery. Next, the posterior bladder ligament and lateral prostate ligament were gradually clamped, cut, and sutured. Finally, the dorsal vascular complex (DVC) and prostate urethral junction was cut and ligated, and the bladder prostate specimen was taken out. When there was no active bleeding on the wound surface, a ureterostomy was performed on the same side of the abdominal wall. Finally, the lower abdominal incision was closed (Fig. 3).

### Outcome measures

The baseline patient parameters and operative characteristics were collected and listed at Table 1. The baseline parameters of patients included age, body mass index (BMI), hemoglobin (Hb), ASA score, tumor staging, transurethral bladder tumor resection (TURBT) pathology, previous abdominal operations, comorbidities (such as hypertension, coronary heart disease, COPD, diabetes mellitus). Operative characteristics involved transfusion needed, estimated blood loss, operation time, resected lymph node, cystectomy pathology, duration of pelvic drainage, and hospital stay after surgery, adjuvant chemotherapy/radiotherapy. The follow-up duration time was also recorded.

The primary outcome measures consisted of complications of patients and survival rate. The complications of patients were recorded, including intraoperative and postoperative complications. Intraoperative complications included bowel, rectal and vascular injuries. Early complications that appeared within 90 days after operation included wound dehiscence, obturator nerve paresis, wound infection, pyelonephritis, deep vein thrombosis, and ileus [14]. Late complications, such as ileus, ureteral stricture, urine leak, lymph leak, and incisional hernia, were those that developed within 90 days or later following operation [14]. The patients’ Clavien classification (CC) of surgical complications was evaluated [12]. Minor complications were those with CC I and II, and major complications were those with CC III and above.

### Follow-up

One month after surgery, patients returned to the clinic for follow-up. Specifically, patients were returned every three months in the first year; every six months in the next year; and once a year later. CT, transabdominal ultrasound and laboratory tests comprised the follow-up investigations. The endpoint of follow-up was December 31, 2022. The recurrence and complications were recorded during follow-up.

### Statistical analyses

The SPSS 22.0 software (SPSS, Chicago, IL, USA) was adopted for data statistics. The count data was presented as a percentage (%). Chi-square test was used for the comparison of the intergroup enumeration data (α = 0.05). The measurement data was displayed as medians and range (Q25, Q75). Kruskal wails H was employed for comparisons among three groups. *P* < 0.05 was regarded to be statistically significant.

## Results

### Comparison of perioperative parameters and follow-up

All patients received RC procedures safely and came back to urologic ward directly after surgery. Perioperative parameters of patients were collected (Table 1). Briefly, there were no significant difference among three groups in BMI, previous abdominal operations, Hb, comorbid conditions, ASA score, clinical stage, and TURBT pathology.

In addition, our results showed that the operation time of LRC (A and B groups) was longer than that of ORC (C group). Compared with C group, the estimated blood loss and transfusion needed in A group and B group were increased. The duration of pelvic drainage was 11.7 days in average in A group, longer than B group (*P* < 0.05) and shorter than C group (*P* < 0.05). The duration of hospital stay in A group was shorter than C group (*P* < 0.05).

Operative specimens of patients in A group were subject to histological examination and draw a conclusion of high grade of muscle-invasive UC. Cancerous tissues were not observed at the edge of the cutting surfaces of ureters, urethral and vas deferens in any patients. No extension of tumors through the prostate and no metastases in regional lymph nodes were detected. Furthermore, more regional lymph nodes were found in A group than C group (*P* < 0.05). Pathological stage showed IN A group, T_2a_N_0_M_0_ was 14 cases, T_2b_N_0_M_0_ was 33 cases, T_3a_N_0_M_0_ was 10 cases.

None of patients in A group received any chemotherapy or radiotherapy postoperatively. All patients were followed up for 3–83 months (61.5 months in average) and were free of cancer relapse. Only 3 patients died of cardiovascular and chronic obstructive diseases until the endpoint of follow-up in A group (Table 1). The OS rates were 94.8% (54/57), 93.7% (59/63), and 29.4% (5/17) in A group, B, and C, respectively. The OS rate in A group was higher than that in C group (*P* < 0.05). The rates of cancer relapse were 0, 3.2% (2/63), and 47.1% (8/17) in the three groups, respectively. The RFS rates were 94.8% (54/57) in A group, 93.7% (59/63) in B group and 23.5% (4/17) in C group. The death rate (BC reason) was lower in A group (0%, 0 case) than C group (41.2%, 7 cases) (*P* < 0.05). The overall death rates was decreased in A group (5.2%, 3 case) compared with C group (70.6%, 12 cases) (*P* < 0.05).

### Comparison of complications

Some complications happened in the three groups (Table 2). In A group, main early complications included ileus (5.2%, 3 cases) and urine leak (5.2%, 3 cases), late complications included ileus (15.8%, 9 cases), pyelonephritis (12.3%, 7 cases) and ureteral stricture (22.8%, 13 cases). In A group, ileus occurred in 12 patients and relapsed in some patients. Compared with C group, the complications rate (including wound dehiscence, wound infection, deep vein thrombosis, and ileus) in A group were decreased (*P* < 0.05), while the complications rate of ureteral stricture was increased (*P* < 0.05). As opposed to B group, the complications rate of ileus and ureteral stricture in A group was increased (*P* < 0.05).

**Table 2:** Comparison of complications of patients in three groups

Parameter	A group (age ≧ 80yrs, LRC)	B group (age < 80yrs, LRC)	C group (age ≧ 80yrs, ORC)	χ^2^	*P*
Bowel	0	0	0		
Rectal	0	0	0		
Iliac vascular	0	0	0		
Ileus	12 (E 3, L 9)	5 (L 5)	15 (E 6, L 9)^*^	18.248	< 0.001
Deep vein thrombosis	1 (E 1)	1 (E 1)	5 (E 5)^*^	13.963	< 0.001
Pyelonephritis	7 (L 7)	8 (L8)	3 (L 3)	0.350	0.839
Wound Infection	2 (E 2)	1 (E 1)	5 (E 5)^*^	12.623	< 0.001
Wound dehiscence	1 (E 1)	0	4 (E 4)^*^	12.762	< 0.001
Ureteral stricture	13 (L 13)	7 (L7)	2 (L 2)	3.065	0.209
Urine Leak	3 (E 3)	2 (E 2)	1 (E 1)	0.842	0.728
Lymph leak	0	0	2 (E 2)^*^	7.162	0.015
Incisional hernia	0	0	0		
Obturator nerve paresis	0	0	0		
Clavien-Classification per Patient:				0.324	0.851
I	39	45	11		
II	18	18	6		

*Abbreviation*: *LRC* Laproscopic radical cystectomy, *ORC* Open radical cystectomy, *E* early (≤ 90 days) postoperatively, *L* late (> 90 days) postoperatively

*P < 0.05 vs. A group

## Discussion

RC in men consists of cystoprostatectomy, urinary diversion and pelvic lymph node resection. There are many complications, and the incidence of these complications can reach 25–35%. The main complications during the operation are bleeding and rectal injury. The amount of bleeding is generally more than 500 ml, up to 2160 ml [15–21]. Researchers reported that the blood loss is much higher in ORC than that in LRC due to the exposure of operation field and limited visual field [22–32]. With the introduction of laparoscopic technology into RC, all details of the operation can be operated in the direct vision field. Such introduction not only provides conditions for fine skills and accurate hemostasis, but also contributes to reducing intraoperative blood loss (ranging between 258 and 791 ml, with an average of about 450 ml) [16–21, 33]. In our study, the average intraoperative blood loss of LRC was less than that in ORC, which is also a strong evidence that laparoscopic technology can reduce intraoperative bleeding.

Laparoscopic technique can reduce intraoperative bleeding, mainly with the help of fine anatomical operation and accurate blood control skills. The main sources of bleeding during RC in men are arteries, veins, a large number of capillary networks in the fatty lymphoid tissue between the lateral wall of the bladder and the pelvis, arteriovenous and capillary vessels between the dorsal side of the bladder and the rectum, DVC and blood vessels in the ligament of the prostate and the bladder.

In the process of LRC of male BC, operation along the gap of oligovascularization can avoid the separation of capillary network in adipose tissue and minimizing diffuse bleeding [34]. The blood supply system of five main arteries for bladder and prostate were isolated and ligated as soon as possible: the inferior bladder artery, the superior bladder artery, the bladder branch of obturator artery and the bladder branch of inferior gluteal artery from the anterior trunk of internal iliac artery. The venous system in or adjacent to the perivesical ligament and the DVC of penis need to be reliably ligated [35]. In particular, DVC mainly contains the superficial branch, deep branch and communicating branch of the dorsal deep vein of the penis, as well as a small amount of arterioles. Therefore, DVC is a significant bleeding site in RC owing to the rich blood vessels and blood flow here.

Controlling bleeding during LRC can reduce the blood loss, thereby protecting the function of important organs through ensuring their oxygen supply. Especially for the octogenarians, the compensatory ability of heart, lung, brain and other important organs is significantly reduced, which is difficult to withstand drastically hemodynamic fluctuations. Massive blood loss is easy to cause heart failure and arrhythmia. Our data showed that the intraoperative blood loss could be controlled below 500 ml in LRC. The blood loss below 500 ml has no negative impact on the oxygen supply and function of important organs. Hence, precise blood control under laparoscope is very important to ensure the life safety of elderly patients undergoing radical resection of bladder cancer.

Health status assessment of oncology patients contributes to lowering the risk and increasing the safety of the RC surgical procedure. The risk of dying from other causes increases with age but not the risk of dying from cancer specifically [36]. For people under 80 years old, RC is linked to the drop of biggest risk in non-disease-related and disease-related death rates [37]. According to data from the National Surgical Quality Improvement Program database, the largest retrospective analysis in septuagenarians and octogenarians with RC (*n* = 1,710) revealed no significant difference in terms of pulmonary, cardiac, or wound complications. In contrast to septuagenarians, octogenarians had a higher mortality risk (4.3% vs. 2.3%) [38]. Despite the fact that chronological age is less significant than frailty, age is still a helpful prognostic indicator for RC [39–41]. Frailty is a syndrome characterized by a diminished capacity to react to stimuli. Frail patients are more likely to die of cancer and suffer from unfavorable side effects from therapy [42]. Octogenarians usually go along with frailty, and assessing quality of life (QoL) and functioning of octogenarian patients is critical [43]. It will be easier to screen patients who benefit from radical surgery and to improve treatment results through stratifying older patients according to frailty [14].

Comorbidity has been linked to poor pathology and survival outcomes after RC [44]. Besides, low pre-operative serum albumin is connected with gastrointestinal (GI) complications and impaired wound healing after RC [9, 45]. Comorbidity assessment aids in the identification of elements that may hinder the treatment of MIBC [46]. The comorbidity, frailty, cognition and anaesthetic risk classification have an impact on the safety of RC. Numerous studies on BC patients have revealed that the Charlson Comorbidity Index (CCI) score is a reliable predictor of CSM [47, 48], overall mortality [49], and peri-operative mortality [37, 50–52]. The age-adjusted CCI, which is simple to calculate, is the most popular comorbidity index for determining long-term survival in cancer [53]. Octogenarian patients benefit from this assessment.

The gastrointestinal peristalsis and secretion function of the elderly are usually weakened, and intestinal dysfunction such as constipation and even incomplete intestinal obstruction often occurs. The recovery of gastrointestinal function of the elderly after operation is much slower than that of the young. Because LRC is performed through abdominal approach, it may interfere with the gastrointestinal function of patients. In order to make the gastrointestinal function of these elderly recovery as soon as possible after operation, we need to pay attention to many aspects. First, the intra-abdominal operation needs to be simplified as much as possible. Although for elderly patients over 80 years old, complex orthotopic neobladder and ileal conduit can also be used for urinary tract diversion, all these operations need to cut the segmental intestine to perform intestinal anastomosis. Intestinal anastomosis can induce complications such as intestinal fistula, anastomotic stenosis, intestinal obstruction and even peritonitis, thereby leading to long-term inability to eat and seriously affecting the recovery of gastrointestinal function. The ureterostomy adopted by this group of patients, rather than the diversion operation of intestinal bladder replacement, is a better choice to simplify the operation. Secondly, ureterostomy can also reduce the incidence of intraperitoneal urinary leakage. Intestinal replacement of bladder is prone to leakage of urine at the anastomosis of ureter and intestinal conduit, causing urinary peritonitis and urinary intestinal paralysis in the abdominal cavity, and delaying the recovery of gastrointestinal function. Thirdly, ureterostomy shortens the overall operation time, reduces the time and dosage of anesthetics, and is conducive to reducing the anesthetic effect on the gastrointestinal tract of the elderly. In this way, the gastrointestinal function of the elderly mostly returns to normal 2 days after operation and can eat independently. Fourth, thorough intestinal preparation before operation can clean up the stool in the intestine, which is conducive to reduce the postoperative flatulence of elderly patients and promote the early peristalsis of the gastrointestinal tract [5]. Fifth, chewing gum, gastrointestinal stimulation with metoclopramide, early mobilisation and oralisation can all shorten the time for the recovery of colon [54]. The “fast tract”/ERAS (Early Recovery After Surgery) regimen has been proven to improve physical and emotional functional scores in patients, as well as reduce the incidence of thrombosis, fever, and wound healing issues [55]. Post-operative pain management, which drastically lowers the usage of opioids and primarily acts as breakthrough pain relievers, is a cornerstone of the ERAS protocol. The majority of patients start taking high-concentration acetaminophen and/or ketorolac intraoperatively rather than epidural opioids and patient-controlled analgesia. Patients on ERAS report higher discomfort than those on a conventional procedure (Visual Analogue Scale 3.1 vs. 1.1, *p* < 0.001), however, ileus after operation reduced from 22 to 7.3% (*p* = 0.003) [56].

The peri-operative mortality of RC was found to be 1.2–3.2% at 30 days and 2.3–8.0% at 90 days in one population-based cohort study and three long-term studies [8, 57–60]. In our study, LRC simplified the operation procedure and reduced the negative impact of surgical trauma on the whole health, the incidence of complications, and the perioperative mortality in octogenarian male patients. Laparoscopic technology is a minimally invasive surgery, which also can reduce the impact of surgical trauma on elderly patients. Our results showed that the patients were able to go to the ground and walk 2–3 days after operation, then recovered and discharged from the hospital 8–11 days postoperatively, suggesting that LRC is suitable and beneficial for elderly patients over 80 years old. Laparoscopic minimally invasive surgery brought less trauma to the patient’s body, the postoperative pain was mild, and there was no need to use analgesics. Besides, laparoscopic surgery and ureterostomy had little interference on intestinal function. Therefore, octogenarian patients can reach rapid recovery of body function and move early, and the occurrence of perioperative mortality can be effectively avoided.

The incidence rate of complications after operation in male octogenarian patients was low and the symptoms were slight in our study. This may be related to the adoption of ureterostomy and the laparoscopic technique. In a sizable single-center cohort, 58% of patients experienced early complications (within 3 months of surgery) [8]. There was a previous study that the type of urinary diversion was typically related to late morbidity [61, 62]. In the first five years of follow-up, 45% of patients had urinary diversion-related complications, and after 15 years of follow-up, this percentage had risen to over 54% [63]. A recent study reported that the overall late complications of RC were 40.2%, and the most common late complications were hydronephrosis (11.6%) and urinary tract infections (20.5%) [64]. There are diverse functional complications, including emptying dysfunction, neobladder continence problems, stoma complications in patients with ileal conduit, stenosis of uretero-intestinal anastomosis, urolithiasis, urinary infections, worsening of renal function, metabolic acidosis, and vitamin B12 deficiency [65]. Neobladder reconstruction is not often advised for patients over 80 years old. Even in high-volume expert centers, orthotopic neobladder surgery is actually infrequently performed in elderly patients (> 80 years) [65]. Actually, the precise age for a severe contraindication remains to be explored to to now. In our study, perioperative main early complications included ileus (5.2%) and urine leak (5.2%). Through analysis, we observed that urinary leakage was related to the decline of growth ability at the anastomosis of skin and ureter in octogenarian patients and the slow healing speed. The leaked urine entered the abdominal cavity along the fissure between the outer edge of ureter and abdominal stoma, resulting in urinary paralytic intestinal obstruction. Moreover, these patients with intestinal obstruction could also excrete a little soft stool and gas, but they had abdominal distension and abdominal pain, belonging to incomplete intestinal obstruction. After the urine leakage disappeared, the intestinal obstruction of these patients was relieved by themselves. Most patients with ileus after 90 days of surgery had the inducement of short-term massive eating. These ileus may be related to the decline of gastrointestinal digestion and emptying ability and varying degrees of intestinal adhesion, which can be alleviated by short-term fasting. In view of this, the octogenarian patients with bladder cancer had better to undergo minimally invasive laparoscopic operation and simple urinary diversion, which can reduce complications, improve physical and physical recovery quickly, and shorten the length of hospital stay. As Faraj K et al. reported that in minimally-invasive cystectomy, with increasing age, hospital stay was notably shorter; and the hospital stay reached 2.56 days in patients aged over 80 years [66].

There are still some limitations in this research. On the one hand, the sample number of patients in this study was small, especially in C group. On the other hand, our research was a retrospective study. Therefore, a larger prospective clinical research is needed to further verify our conclusions.

## Conclusion

In summary, LRC combined with bilateral cutaneous ureterostomy is safe, feasible and better selection for the treatment of male octogenarian patients with MIBC. The octogenarian receiving cutaneous ureterostomy recovers slowly and exists a rate of incomplete intestinal obstruction (21%) postoperatively.

## Data Availability

The datasets used and/or analyzed during the current study are available from the corresponding author on reasonable request.

## Abbreviations

LRC: Laproscopic radical cystectomy
ORC: Open radical cystectomy
Hb: Hemoglobin
BMI: Body Mass Index
COPD: Chronic obstructive pulmonary diseases
ASA: American Society of Anesthesiologists
TURBT: Transurethral bladder tumor resection
BC: Bladder cancer
